# Computational Modeling and Parametric Analysis of SMA Hybrid Composite Plates under Thermal Environment

**DOI:** 10.3390/s23031344

**Published:** 2023-01-25

**Authors:** Wei Li, Ivo Stachiv

**Affiliations:** Institute of Physics, Czech Academy of Sciences, Na Slovance 2, 18200 Prague, Czech Republic

**Keywords:** shape memory alloys, SMA, first-order deformation theory, composite laminated structures, FEM, computational modeling

## Abstract

This paper presents a coupled thermoelastic finite element formulation for static and dynamic analysis of composite laminated plates with embedded active shape memory alloy (SMA) wires, which accounts for both the phase transformation and the nonlinearity effects of SMA wires. The equations of motion are obtained by using Hamilton’s principle and first-order shear deformation theory (FSDT). Furthermore, based on Brinson’s one-dimensional phase transformation constitutive law, a novel coupled thermoelastic finite element model that enables analysis of the SMA hybrid composite (SMAHC) plate is developed. The accuracy and efficiency of the developed computational model for analysis of SMAHC plates are reinforced by comparing theoretical predictions with data available from the literature. The results of the numerical examples also show the ability of the proposed model to predict the thermal-mechanical behavior of SMAHC plates in accordance with SMA’s hysteresis behavior. In addition, based on the proposed model, the influence of temperature as well as SMA volume fraction, pre-strain value, boundary condition and layup sequence on the static bending and free vibration behavior of the SMAHC plates is investigated in detail. The results of parametric analysis show that the variations of both static deflection and natural frequency of the SMAHC plate over temperature exhibit a nonmonotonic behavior.

## 1. Introduction

Over the past decades, numerous studies have been conducted on intelligent materials to improve the structural performance of various sensors and actuators. Shape memory alloys (SMAs), due to their extremely large, recoverable strains and unusual solid-state phase transformation properties, have broad applications as active components of adaptive structures. For example, SMA elements can be embedded into the laminated composite structures to create smart sensing and actuating devices. Hence, nowadays, the SMA-based hybrid laminated composite structures are widely used for innovative applications in various areas including aerospace [1], automotive [2] and civil engineering [3].

Among the scientific research into smart SMA-based hybrid composites (SMAHC) in particular, the pioneering researchers mainly focused on the mechanical behavior modifications of SMAHC structures. The concept of embedding SMAs into a composite medium was first proposed by Rogers et al. [4,5]. In these studies, the SMAs were used to improve the vibrational and buckling behaviors of the designed composite. Later, extensive research was performed to investigate active control of SMA-based hybrid composite beams [6,7,8,9], plates [10,11,12,13,14,15], shell panels [16] and sandwich panels [17,18]. More recently, due to the wide application of SMAHC structures in engineering, theoretical frameworks, which are qualified for accurately analyzing their dynamic and static behaviors, have drawn extensive attention. Lu et al. [19] presented an analytical method for bending SMA fiber-reinforced laminated beams. The analytical formulations used to investigate the thermal buckling and vibrational behavior of SMA hybrid laminated composite beams [20] and plates [21] were also developed. Kabir and Tehrani [22] performed analytical investigations of thermo-mechanical buckling and postbuckling of symmetric SMAHC plates, and the influence of SMA activation temperature, SMA volume fraction and pre-strain on thermo-mechanical buckling and postbuckling behavior were also discussed. A closed-form solution was presented by Bayat and Toussi [23] to analyze the thermal buckling and postbuckling behavior of SMA wire-reinforced laminated composite beams. Fahim et al. [24] developed a semianalytical model to investigate the bending response of SMA composite beams.

Although it is well acknowledged that the analytical solutions are more favorable and convenient for parametric investigation, the finite element method (FEM), on the other hand, provides a more general solution for modeling complex geometrical structures and boundary conditions. Thermal stability and vibration behavior of SMA hybrid beams [25], plates [26,27,28], shell panels [29,30,31,32] and sandwich panels [33] were studied by using FEM based on various plate/shell theories. Ghomshei et al. [34] proposed a nonlinear FEM for thick composite beams with embedded SMA ribbons or wires by using a higher-order shear deformation beam theory and the von-Karman nonlinear strains. Cho and Rhee [35] presented a nonlinear finite element approach to study the bending of a cantilevered SMA wire-reinforced composite shell subjected to both structural and thermal loads. Recently, static and dynamic analyses of composite plates were performed based on FSDT [36,37,38], and Tabrizikahou et al. [39] used FEM to investigate out-of-plane behavior of masonry prisms retrofitted with SMA stripes.

Surprisingly, the study of a composite laminated plate embedded with active SMA wires under thermal environment has not yet been reported. In this study, a coupled thermoelastic FEM for static and dynamic analysis of SMAHC plates is developed based on the first-order shear deformation theory. The governing equations are then obtained by using Hamilton’s principle. Compared to previous research, the phase transformation and nonlinearity effects of SMA are included in the developed method based on the one-dimensional Brinson model, and both thermal and recovery stresses are also reported for the constitutive relation of SMAHC lamina. We emphasize here that the present model is directly applicable to the investigation of other elastic beams or plates with different geometries, material layers and boundary conditions.

This paper is organized as follows. In Section 2, constitutive relations of SMA materials based on Brinson’s model are briefly presented, and thermo-mechanical properties of an SMA hybrid lamina are further derived by using a micro/macro mixture method. Then, a coupled thermoelastic finite element formulation for SMAHC plates based on FSDT plate theory is developed. To demonstrate the accuracy of the proposed FEM model, a cantilevered SMAHC plate is investigated in Section 3, and both static response and free vibration results are compared to previously published results. Section 3 also presents a detailed parametric investigation to study the influences of SMA volume fraction, pre-strain level, boundary condition and layup sequence on the static and thermal vibration behavior of the SMAHC plates. Finally, the conclusions are presented in last section.

## 2. Theoretical Model

### 2.1. Constitutive Relation of SMA

The present work employs Brinson’s one-dimensional constitutive equation of SMA for the modeling of SMA hybrid layers. The constitutive relation of Brinson [40] governs the stress (*σ*) to the strain (*ε*), temperature (*T*) and martensitic fraction (*ξ*) in the following form:

(1) $$\sigma -\sigma_{0}=E\left(\xi \right)\left(\varepsilon \right)-E\left(\xi_{0}\right)\left(\varepsilon_{0}\right)+\Omega \left(\xi \right)\left(\xi_{s}\right)-\Omega \left(\xi_{0}\right)\left(\xi_{s0}\right)+\theta \left(T-T_{0}\right),$$

where E(ξ), θ and Ω(ξ) stand for the Young’s modulus, thermoelastic tensor and phase transformation tensor, respectively. The subscript “0” indicates the initial conditions. In addition, E(ξ) and Ω(ξ) can be defined as:

(2) $$E\left(\xi \right)=E_{A}+\xi \left(E_{M}-E_{A}\right),$$

(3) $$\Omega \left(\xi \right)=-\varepsilon_{L}E\left(\xi \right),$$

where $E_{A}$ and $E_{M}$ are the Young’s modulus of SMA in the austenite and martensite state, respectively; $\xi =\xi_{s}+\xi_{T}$ is the martensite volume fraction which is divided into the stress-induced martensite and temperature-induced martensite; and $\varepsilon_{L}$ is the maximum recoverable strain. Detailed kinetic equations of phase transformation are given in Appendix A, and the expressions of elastic modulus of an SMA hybrid lamina are presented in Appendix B.

### 2.2. Equation of Motion

**Figure 1:**
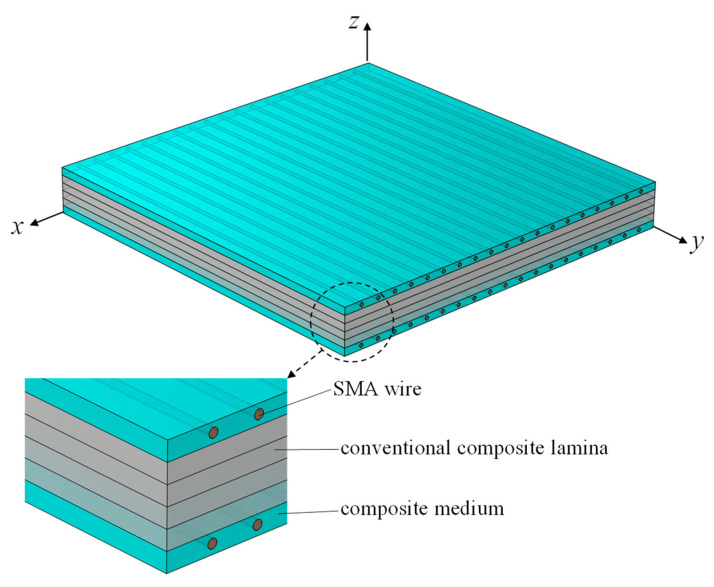
The sketch of the considered SMAHC plate and its coordinate system.

It is assumed that the considered SMA hybrid composite laminated plate has length *a*, width *b* and total thickness *h*, as shown in Figure 1. The hybrid plate consists of conventional composite lamina and SMA wire-reinforced hybrid composite lamina. Based on FSDT plate theory [41], the displacement field of an SMAHC plate can be expressed as:

(4a) $$u=u_{0}\left(x,y,t\right)+z\phi_{x}\left(x,y,t\right),$$

(4b) $$v=v_{0}\left(x,y,t\right)+z\phi_{y}\left(x,y,t\right),$$

(4c) $$w=w_{0}\left(x,y,t\right),$$

where (*u*, *v*, *w*) denote the plate displacement vectors in *x*, *y* and *z* directions, and the subscript “0” stands for displacement of the mid-plate components of the plate; $\phi_{x}$ and $\phi_{y}$ represent rotation terms around *y* and *x* axes.

The strain-displacement relations can be determined as follows:

(5) $$\left\{\begin{array}{c} \varepsilon_{xx}\\ \varepsilon_{yy}\\ \begin{array}{c} \gamma_{yz}\\ \gamma_{xz}\\ \gamma_{xy} \end{array} \end{array}\right\}=\left\{\begin{array}{c} \varepsilon_{xx}^{m}\\ \varepsilon_{yy}^{m}\\ \begin{array}{c} \gamma_{yz}^{m}\\ \gamma_{xz}^{m}\\ \gamma_{xy}^{m} \end{array} \end{array}\right\}+z\left\{\begin{array}{c} \varepsilon_{xx}^{b}\\ \varepsilon_{yy}^{b}\\ \begin{array}{c} \gamma_{yz}^{b}\\ \gamma_{xz}^{b}\\ \gamma_{xy}^{b} \end{array} \end{array}\right\}=\left\{\begin{array}{c} u_{0,x}\\ v_{0,y}\\ \begin{array}{c} w_{0,y}+\phi_{y}\\ w_{0,x}+\phi_{x}\\ u_{0,y}+v_{0,x} \end{array} \end{array}\right\}+\left\{\begin{array}{c} \phi_{x,x}\\ \phi_{y,y}\\ \begin{array}{c} 0\\ 0\\ \phi_{x,y}+\phi_{y,x} \end{array} \end{array}\right\},$$

where the superscripts “*m*” and “*b*” represent the in-plane and out-of-plane terms, and the subscript “,” stands for a differential calculation.

For an SMAHC plate subjected to a uniform temperature rise, the constitutive law of *k*th lamina of the plate can be expressed as:

(6) $${\left\{\begin{array}{c} \begin{array}{c} \sigma_{xx}\\ \sigma_{yy}\\ \sigma_{zz} \end{array}\\ \begin{array}{c} \sigma_{yz}\\ \sigma_{xz}\\ \sigma_{xy} \end{array} \end{array}\right\}}^{k}={\left[\begin{array}{llllll} {\bar{C}}_{11} & {\bar{C}}_{12} & {\bar{C}}_{13} & 0 & 0 & {\bar{C}}_{16}\\ {\bar{C}}_{16} & {\bar{C}}_{22} & {\bar{C}}_{23} & 0 & 0 & {\bar{C}}_{26}\\ {\bar{C}}_{13} & {\bar{C}}_{23} & {\bar{C}}_{33} & 0 & 0 & {\bar{C}}_{36}\\ 0 & 0 & 0 & {\bar{C}}_{44} & {\bar{C}}_{45} & 0\\ 0 & 0 & 0 & {\bar{C}}_{45} & {\bar{C}}_{55} & 0\\ {\bar{C}}_{16} & {\bar{C}}_{26} & {\bar{C}}_{36} & 0 & 0 & {\bar{C}}_{66} \end{array}\right]}^{k}{\left(\left\{\begin{array}{c} \varepsilon_{xx}\\ \varepsilon_{yy}\\ \begin{array}{c} \varepsilon_{zz}\\ \gamma_{yz}\\ \begin{array}{c} \gamma_{xz}\\ \gamma_{xy} \end{array} \end{array} \end{array}\right\}-\Delta T\left\{\begin{array}{c} \alpha_{xx}\\ \alpha_{yy}\\ \begin{array}{c} 0\\ 0\\ \begin{array}{c} 0\\ 2\alpha_{xy} \end{array} \end{array} \end{array}\right\}\right)}^{k}+V_{s}{\left\{\begin{array}{c} \sigma_{xx}^{r}\\ \sigma_{yy}^{r}\\ \begin{array}{c} 0\\ 0\\ \begin{array}{c} 0\\ \sigma_{xy}^{r} \end{array} \end{array} \end{array}\right\}}^{k}$$

Equation (6) can then be rewritten in a compact form as:

(7) $${\left\{\sigma \right\}}^{k}={\left[\bar{C}\right]}^{k}{\left\{\varepsilon -\Delta T\alpha \right\}}^{k}+V_{s}{\left\{\sigma^{r}\right\}}^{k}$$

where $\left[{\bar{C}}_{ij}\right]$ is the transformed reduced stiffness, $\left\{\alpha_{ij}\right\}$ are the thermal expansion coefficients and $\left\{\sigma_{ij}^{r}\right\}$ represent temperature and pre-strain value-dependent recovery stress obtained from Equation (1). It should be noted that the constitutive relation shown in Equation (6) is not only applicable to an SMA hybrid layer but also to the conventional composite layers by setting the recovery stress term $\left\{\sigma_{ij}^{r}\right\}$ equal to zero. The in-plane force, bending moment, transverse force, thermal force and recovery stress resultants of the SMA hybrid plate are defined as:

(8a) $$\left\{N\right\}=\left[\begin{array}{ccc} N_{xx} & N_{yy} & N_{xy} \end{array}\right]=\int_{-h/2}^{h/2}\left[\begin{array}{ccc} \sigma_{xx} & \sigma_{yy} & \sigma_{xy} \end{array}\right]dz,$$

(8b) $$\left\{M\right\}=\left[\begin{array}{ccc} M_{xx} & M_{yy} & M_{xy} \end{array}\right]=\int_{-h/2}^{h/2}z\cdot \left[\begin{array}{ccc} \sigma_{xx} & \sigma_{yy} & \sigma_{xy} \end{array}\right]dz,$$

(8c) $$\left\{Q\right\}=\left[\begin{array}{cc} Q_{x} & Q_{y} \end{array}\right]=\int_{-h/2}^{h/2}\left[\begin{array}{cc} \sigma_{xz} & \sigma_{yz} \end{array}\right]dz.$$

Combining the constitutive relations given by Equation (6) with Equation (8) yields:

(9a) $$\left\{N\right\}=\left[A\right]\left\{\varepsilon^{m}\right\}+\left[B\right]\left\{\varepsilon^{b}\right\}-\left\{N^{T}\right\}+\left\{N^{r}\right\},$$

(9b) $$\left\{M\right\}=\left[B\right]\left\{\varepsilon^{m}\right\}+\left[D\right]\left\{\varepsilon^{b}\right\}-\left\{M^{T}\right\}+\left\{M^{r}\right\},$$

(9c) $$\left\{Q\right\}=\left[A\right]\left\{\varepsilon^{m}\right\},$$

where the extensional stiffness matrix [A], coupling matrix [B] and bending stiffness matrix [D] of the plate are given as follows:

(10) $$A_{ij}=\sum_{k=1}^{N_{L}}{\left[{\bar{C}}_{ij}\right]}^{k}\left(h_{k}-h_{k-1}\right),\;B_{ij}=\frac{1}{2}\sum_{k=1}^{N_{L}}{\left[{\bar{C}}_{ij}\right]}^{k}\left(h_{k}^{2}-h_{k-1}^{2}\right),\;D_{ij}=\frac{1}{3}\sum_{k=1}^{N_{L}}{\left[{\bar{C}}_{ij}\right]}^{k}\left(h_{k}^{3}-h_{k-1}^{3}\right).$$

where *N*_L_ stands for the number of layers.

(11a) $$\left\{N^{T}\right\}=\sum_{k=1}^{N_{L}}{\left[\bar{C}\right]}^{k}{\left\{\alpha \right\}}^{k}\Delta T\left(h_{k}-h_{k-1}\right),\;\left\{M^{T}\right\}=\frac{1}{2}\sum_{k=1}^{N_{L}}{\left[\bar{C}\right]}^{k}{\left\{\alpha \right\}}^{k}\Delta T\left(h_{k}^{2}-h_{k-1}^{2}\right),$$

(11b) $$\left\{N^{r}\right\}=\sum_{k=1}^{N_{L}}{\left[\bar{C}\right]}^{k}V_{s}{\left\{\sigma^{r}\right\}}^{k}\left(h_{k}-h_{k-1}\right),\left\{M^{r}\right\}=\frac{1}{2}\sum_{k=1}^{N_{L}}{\left[\bar{C}\right]}^{k}V_{s}{\left\{\sigma^{r}\right\}}^{k}\left(h_{k}^{2}-h_{k-1}^{2}\right).$$

Note that the coupling matrix [B] and recovery stress bending resultant $M^{r}$ will not be present for the symmetric plates. The equations of motion for an SMAHC plate can be obtained according to the FSDT in conjunction with Hamilton’s principle. Mathematically, it is given by [42,43] as:

(12) $$\int_{0}^{T}\left(\delta U+\delta W-\delta K\right)dt=0,$$

where δU, δW and δK refer to the virtual elastic potential energy, the virtual work performed by external mechanical and/or thermal loads and the virtual kinetic energy of the SMAHC plate, respectively. Namely, each term can be expressed as:

(13a) $$\delta U=\int_{A}^{}\left\{\int_{-\frac{h}{2}}^{\frac{h}{2}}\sigma_{ij}\left(\delta \varepsilon_{ij}^{m}+z\delta \varepsilon_{ij}^{b}\right)dz\right\}dxdy,$$

(13b) $$\delta W=-\left(\int_{\Omega }^{}q_{z}\delta w_{0}dA+\int_{\Gamma }^{}P_{x}ds+\int_{\Gamma }^{}P_{y}ds+\int_{\Gamma }^{}T_{x}\delta w_{0,x}^{}ds+\int_{\Gamma }^{}T_{y}\delta w_{0,y}^{}ds\right),$$

(13c) $$\begin{array}{l} \delta K=\int_{\Omega }^{}\sum_{k=1}^{N_{L}}\int_{h_{k}}^{}\rho_{k}[\left({\dot{u}}_{0}+z{\dot{\phi }}_{x}\right)\left(\delta {\dot{u}}_{0}+z\delta {\dot{\phi }}_{x}\right)+\left({\dot{v}}_{0}+z{\dot{\phi }}_{y}\right)\left(\delta {\dot{v}}_{0}+z\delta {\dot{\phi }}_{y}\right)\\ \;\;\;\;\;\;\;\;\;\;\;\;\;+{\dot{w}}_{0}\delta {\dot{w}}_{0}]dzdxdy, \end{array}$$

where $q_{z}$ is the force along the *z* axis; $h_{k}$ is the thickness of *k*th layer; $\rho_{k}\;$is the mass density of the *k*th layer material; and a superposed dot on a variable indicates its time derivative. $P_{x},\;P_{y},\;T_{x}\;$and $T_{y}$ are the boundary terms, which are listed in Appendix C.

Then, by integration by parts to weaken the differentiability of *u*_0_, *v*_0_ and *w*_0_, the Euler–Lagrange equations of motion are obtained as below.

(14) $$\begin{array}{cc} \delta u_{0}: & N_{xx,x}+N_{xy,y}=I_{0}{\ddot{u}}_{0}+I_{1}{\ddot{\phi }}_{x},\\ \delta v_{0}: & N_{xy,x}+N_{yy,y}=I_{0}{\ddot{v}}_{0}+I_{1}{\ddot{\phi }}_{y},\\ \delta w_{0}: & Q_{xz,x}+Q_{yz,y}+N_{xx}w_{0,xx}+N_{yy}w_{0,yy}+2N_{xy}w_{0,xy}+q_{z}=I_{0}{\ddot{w}}_{0},\\ \delta \phi_{x}: & M_{xx,x}+M_{xy,y}-Q_{xz}=I_{1}{\ddot{u}}_{0}+I_{2}{\ddot{\phi }}_{x},\\ \delta \phi_{y}: & M_{xy,x}+M_{yy,y}-Q_{yz}=I_{1}{\ddot{v}}_{0}+I_{2}{\ddot{\phi }}_{y}.\; \end{array}$$

where the mass moments of inertia are given by:

(15) $$\left[\begin{array}{ccc} I_{0} & I_{1} & I_{2} \end{array}\right]=\sum_{k=1}^{N_{L}}\int_{h_{k}}^{}\rho_{k}\left[\begin{array}{ccc} 1 & z & z^{2} \end{array}\right]\mathrm{dz},$$

### 2.3. Finite Element Method—Problem Formulation

It is evident from structure of Equation (15) that the displacement components of each node can be further discretized at *k*th layer and, correspondingly, used to obtain the required finite element model of the SMAHC plates. The generalized displacements are approximated by using the following interpolations [41]:

(16) $$\begin{array}{c} u_{0}^{e}\left(x,y,t\right)=\overset{N_{e}}{\underset{j=1}{\sum }}u_{j}^{e}\left(t\right)\psi_{j}^{e}\left(x,y\right),\\ v_{0}^{e}\left(x,y,t\right)=\overset{N_{e}}{\underset{j=1}{\sum }}v_{j}^{e}\left(t\right)\psi_{j}^{e}\left(x,y\right),\\ w_{0}^{e}\left(x,y,t\right)=\overset{N_{e}}{\underset{j=1}{\sum }}w_{j}^{e}\left(t\right)\psi_{j}^{e}\left(x,y\right),\\ \phi_{x}^{e}\left(x,y,t\right)=\overset{N_{e}}{\underset{j=1}{\sum }}\Phi^{1}\left(t\right)\psi_{j}^{e}\left(x,y\right),\\ \phi_{y}^{e}\left(x,y,t\right)=\overset{N_{e}}{\underset{j=1}{\sum }}\Phi^{2}\left(t\right)\psi_{j}^{e}\left(x,y\right). \end{array}$$

Here, “*e*” stands for element, and “$N_{e}$” means the number of nodes in the element.

Substituting Equation (16) for $\left(u_{0},v_{0},w_{0},\phi_{x},\phi_{y}\;\right)$ into the Euler-Lagrange equations (see Equation (14)), the FEM of SMAHC plates based on FSDT can be obtained in the following way:

(17) $$\begin{array}{l} \left[\begin{array}{ccccc} \left[M_{0}\right] & 0 & 0 & \left[M_{1}\right] & 0\\ 0 & \left[M_{0}\right] & 0 & 0 & \left[M_{1}\right]\\ 0 & 0 & \left[M_{0}\right] & 0 & 0\\ \left[M_{1}\right] & 0 & 0 & \left[M_{2}\right] & 0\\ 0 & \left[M_{1}\right] & 0 & 0 & \left[M_{2}\right] \end{array}\right]\left\{\begin{array}{c} \left\{{\ddot{u}}^{e}\right\}\\ \left\{{\ddot{v}}^{e}\right\}\\ \left\{{\ddot{w}}^{e}\right\}\\ \left\{{\ddot{\Phi }}^{1}\right\}\\ \left\{{\ddot{\Phi }}^{2}\right\} \end{array}\right\}+\left[\begin{array}{ccccc} \left[K_{11}\right] & \left[K_{12}\right] & \left[K_{13}\right] & \left[K_{14}\right] & \left[K_{15}\right]\\ {\left[K_{12}\right]}^{T} & \left[K_{22}\right] & \left[K_{23}\right] & \left[K_{24}\right] & \left[K_{25}\right]\\ {\left[K_{13}\right]}^{T} & {\left[K_{23}\right]}^{T} & \left[K_{33}\right] & \left[K_{34}\right] & \left[K_{35}\right]\\ {\left[K_{14}\right]}^{T} & {\left[K_{24}\right]}^{T} & {\left[K_{34}\right]}^{T} & \left[K_{44}\right] & \left[K_{45}\right]\\ {\left[K_{15}\right]}^{T} & {\left[K_{25}\right]}^{T} & {\left[K_{35}\right]}^{T} & {\left[K_{45}\right]}^{T} & \left[K_{55}\right] \end{array}\right]\left\{\begin{array}{c} \begin{array}{c} \left\{u^{e}\right\}\\ \left\{v^{e}\right\}\\ \left\{w^{e}\right\} \end{array}\\ \left\{\Phi^{1}\right\}\\ \left\{\Phi^{2}\right\} \end{array}\right\}\\ \;\;\;\;\;\;\;\;\;\;\;\;=\left[\begin{array}{c} \begin{array}{c} \left\{F^{1}\right\}\\ \left\{F^{2}\right\}\\ \left\{F^{3}\right\} \end{array}\\ \begin{array}{c} \left\{F^{4}\right\}\\ \left\{F^{5}\right\} \end{array} \end{array}\right]-\left[\begin{array}{c} \begin{array}{c} \left\{F^{T1}\right\}\\ \left\{F^{T2}\right\}\\ \left\{0\right\} \end{array}\\ \begin{array}{c} \left\{F^{T4}\right\}\\ \left\{F^{T5}\right\} \end{array} \end{array}\right]+\left[\begin{array}{c} \begin{array}{c} \left\{F^{r1}\right\}\\ \left\{F^{r2}\right\}\\ \left\{0\right\} \end{array}\\ \begin{array}{c} \left\{F^{r4}\right\}\\ \left\{F^{r5}\right\} \end{array} \end{array}\right] \end{array}$$

In the case of the static response analysis, Equation (17) can be reduced as:

(18) $$\left[\begin{array}{ccccc} \left[K_{11}\right] & \left[K_{12}\right] & \left[K_{13}\right] & \left[K_{14}\right] & \left[K_{15}\right]\\ {\left[K_{12}\right]}^{T} & \left[K_{22}\right] & \left[K_{23}\right] & \left[K_{24}\right] & \left[K_{25}\right]\\ {\left[K_{13}\right]}^{T} & {\left[K_{23}\right]}^{T} & \left[K_{33}\right] & \left[K_{34}\right] & \left[K_{35}\right]\\ {\left[K_{14}\right]}^{T} & {\left[K_{24}\right]}^{T} & {\left[K_{34}\right]}^{T} & \left[K_{44}\right] & \left[K_{45}\right]\\ {\left[K_{15}\right]}^{T} & {\left[K_{25}\right]}^{T} & {\left[K_{35}\right]}^{T} & {\left[K_{45}\right]}^{T} & \left[K_{55}\right] \end{array}\right]\left\{\begin{array}{c} \begin{array}{c} \left\{u^{e}\right\}\\ \left\{v^{e}\right\}\\ \left\{w^{e}\right\} \end{array}\\ \left\{\Phi^{1}\right\}\\ \left\{\Phi^{2}\right\} \end{array}\right\}=\left[\begin{array}{c} \begin{array}{c} \left\{F^{1}\right\}\\ \left\{F^{2}\right\}\\ \left\{F^{3}\right\} \end{array}\\ \begin{array}{c} \left\{F^{4}\right\}\\ \left\{F^{5}\right\} \end{array} \end{array}\right]-\left[\begin{array}{c} \begin{array}{c} \left\{F^{T1}\right\}\\ \left\{F^{T2}\right\}\\ \left\{0\right\} \end{array}\\ \begin{array}{c} \left\{F^{T4}\right\}\\ \left\{F^{T5}\right\} \end{array} \end{array}\right]+\left[\begin{array}{c} \begin{array}{c} \left\{F^{r1}\right\}\\ \left\{F^{r2}\right\}\\ \left\{0\right\} \end{array}\\ \begin{array}{c} \left\{F^{r4}\right\}\\ \left\{F^{r5}\right\} \end{array} \end{array}\right].$$

Finally, in the case of the free vibrations analysis under thermal environment (i.e., natural frequencies), Equation (17) yields:

(19) $$\left(\left[\begin{array}{ccccc} \left[K_{11}\right] & \left[K_{12}\right] & \left[K_{13}\right] & \left[K_{14}\right] & \left[K_{15}\right]\\ {\left[K_{12}\right]}^{T} & \left[K_{22}\right] & \left[K_{23}\right] & \left[K_{24}\right] & \left[K_{25}\right]\\ {\left[K_{13}\right]}^{T} & {\left[K_{23}\right]}^{T} & \left[K_{33}\right] & \left[K_{34}\right] & \left[K_{35}\right]\\ {\left[K_{14}\right]}^{T} & {\left[K_{24}\right]}^{T} & {\left[K_{34}\right]}^{T} & \left[K_{44}\right] & \left[K_{45}\right]\\ {\left[K_{15}\right]}^{T} & {\left[K_{25}\right]}^{T} & {\left[K_{35}\right]}^{T} & {\left[K_{45}\right]}^{T} & \left[K_{55}\right] \end{array}\right]-\omega^{2}\left[\begin{array}{cc} \begin{array}{cc} \begin{array}{c} \begin{array}{c} \left[M_{0}\right]\\ 0\\ 0 \end{array}\\ \left[M_{1}\right]\\ 0 \end{array} & \begin{array}{c} \begin{array}{c} 0\\ \left[M_{0}\right]\\ 0 \end{array}\\ 0\\ \left[M_{1}\right] \end{array} \end{array} & \begin{array}{ccc} \begin{array}{c} \begin{array}{c} 0\\ 0\\ \left[M_{0}\right] \end{array}\\ 0\\ 0 \end{array} & \begin{array}{c} \begin{array}{c} \left[M_{1}\right]\\ 0\\ 0 \end{array}\\ \left[M_{2}\right]\\ 0 \end{array} & \begin{array}{c} \begin{array}{c} 0\\ \left[M_{1}\right]\\ 0 \end{array}\\ 0\\ \left[M_{2}\right] \end{array} \end{array} \end{array}\right]\right)\left\{\begin{array}{c} \begin{array}{c} \left\{u^{e}\right\}\\ \left\{v^{e}\right\}\\ \left\{w^{e}\right\} \end{array}\\ \left\{\Phi^{1}\right\}\\ \left\{\Phi^{2}\right\} \end{array}\right\}=\left[\begin{array}{c} \begin{array}{c} \left\{F^{r1}\right\}\\ \left\{F^{r2}\right\}\\ \left\{0\right\} \end{array}\\ \begin{array}{c} \left\{F^{r4}\right\}\\ \left\{F^{r5}\right\} \end{array} \end{array}\right]-\left[\begin{array}{c} \begin{array}{c} \left\{F^{T1}\right\}\\ \left\{F^{T2}\right\}\\ \left\{0\right\} \end{array}\\ \begin{array}{c} \left\{F^{T4}\right\}\\ \left\{F^{T5}\right\} \end{array} \end{array}\right],$$

where ω is the natural frequency of free vibration of the SMAHC plate. $\left(M_{0},M_{1},M_{2}\right)$ are the mass matrix, $K_{ij}$ represents the global stiffness matrix and $\left(F^{i},F^{Ti},F^{ri}\right)$ are the mechanical force, thermal force, and SMA recovery force, respectively. The detailed expressions of these submatrices are listed in Appendix C.

### 2.4. Solution Algorithm

To solve the finite element formulation of Equation (17), the Newmark method is applied for the incremental time integration with respect to the SMA phase transformation process. First, we rewrite Equation (17) in a symbolic form as:

(20) $$\left[M^{e}\right]\left\{{\ddot{\Delta }}^{e}\left(t\right)\right\}+\left[K^{e}\right]\left\{\Delta^{e}\left(t\right)\right\}=\left\{F^{e}\left(t,\xi \right)\right\},$$

where $\Delta^{e}$ is the generalized element displacement vector, and the superposed dot indicates time derivative. According to Newmark’s time integration scheme [44], the displacement vector and its time derivatives can be expressed as:

(21a) $${\left\{\Delta^{e}\right\}}_{t+1}={\left\{\Delta^{e}\right\}}_{t}+\Delta t{\left\{{\dot{\Delta }}^{e}\right\}}_{t}+\frac{1}{2}\Delta t^{2}{\left\{{\ddot{\Delta }}^{e}\right\}}_{t+\gamma },$$

(21b) $${\left\{{\dot{\Delta }}^{e}\right\}}_{t+1}={\left\{{\dot{\Delta }}^{e}\right\}}_{t}+\Delta t{\left\{{\ddot{\Delta }}^{e}\right\}}_{t+\alpha },$$

(21c) $${\left\{{\ddot{\Delta }}^{e}\right\}}_{t+\alpha }=\left(1-\alpha \right){\left\{{\ddot{\Delta }}^{e}\right\}}_{t}+\alpha {\left\{{\ddot{\Delta }}^{e}\right\}}_{t+1},$$

where $\Delta^{e}$ is the generalized element displacement vector. α and γ are the stability and accuracy parameters, and in this study, the constant average acceleration scheme (α=γ=0.5) is taken for time integration.

By using Equation (21), the finite element equation in Equation (20) can be discretized as:

(22) $${\left[{\hat{K}}^{e}\right]}_{t+1}{\left\{\Delta^{e}\right\}}_{t+1}={\left\{{\hat{F}}^{e}\right\}}_{t,t+1},$$

where:

(23) $${\left[{\hat{K}}^{e}\right]}_{t+1}={\left[K^{e}\right]}_{t+1}+\frac{4}{\Delta t^{2}}{\left[M^{e}\right]}_{t+1},$$

(24) $${\left[{\hat{F}}^{e}\right]}_{t,t+1}={\left[F^{e}\right]}_{t+1}+{\left[M^{e}\right]}_{t+1}\left(\frac{4}{\Delta t^{2}}{\left\{\Delta^{e}\right\}}_{t}+\frac{4}{\Delta t}{\left\{{\dot{\Delta }}^{e}\right\}}_{t}+{\left\{{\ddot{\Delta }}^{e}\right\}}_{t}\right).$$

At the end of each incremental time step, the velocity and acceleration vectors are updated by using the following equations:

(25) $${\left\{{\dot{\Delta }}^{e}\right\}}_{t+1}={\left\{{\dot{\Delta }}^{e}\right\}}_{t}+\frac{\Delta t}{2}{\left\{{\ddot{\Delta }}^{e}\right\}}_{t}+\frac{\Delta t}{2}{\left\{{\ddot{\Delta }}^{e}\right\}}_{t+1},$$

(26) $${\left\{{\ddot{\Delta }}^{e}\right\}}_{t+1}=\frac{4}{\Delta t^{2}}\left({\left\{\Delta^{e}\right\}}_{t+1}-{\left\{\Delta^{e}\right\}}_{t}\right)-\frac{4}{\Delta t}{\left\{{\dot{\Delta }}^{e}\right\}}_{t}-{\left\{{\ddot{\Delta }}^{e}\right\}}_{t}.$$

**Figure 2:**
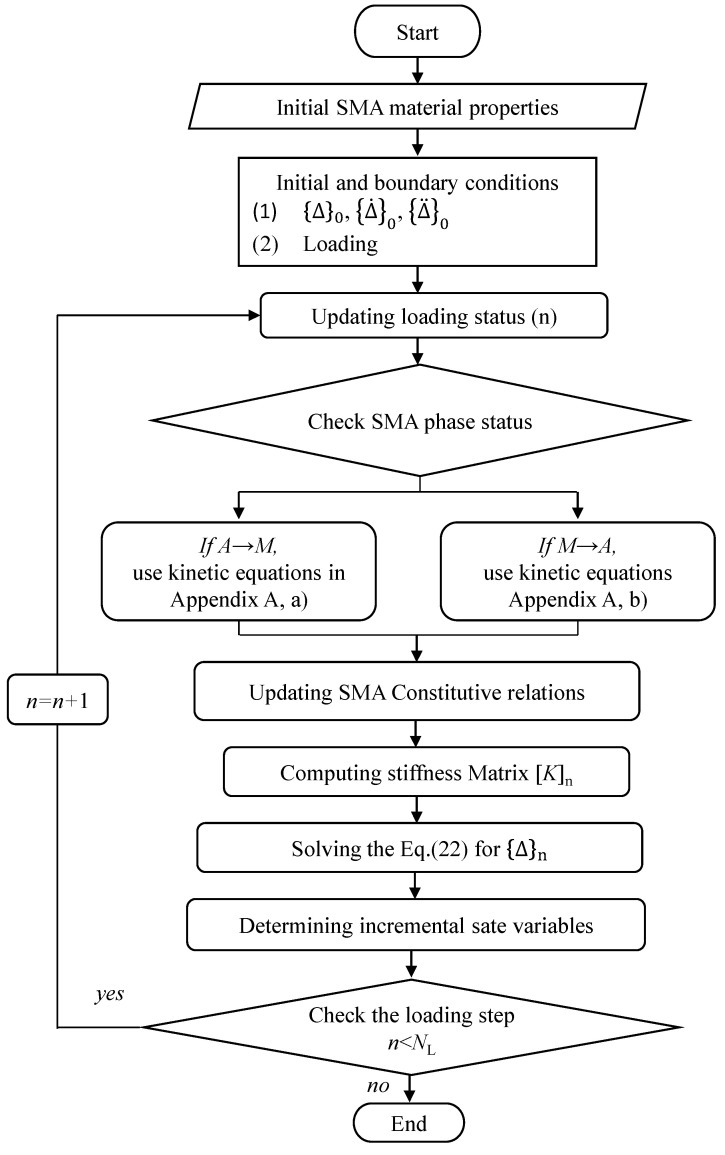
Solution algorithm for the SMAHC plate.

The flow chart of the presented step-by-step solution scheme is shown in Figure 2.

## 3. Results and Discussion

### 3.1. Validation of Developed Model

**Figure 3:**
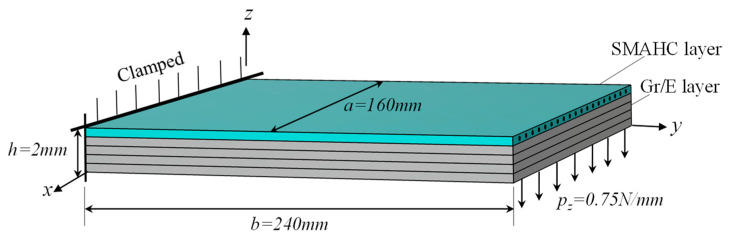
Considered geometry, loading and boundary conditions of the SMAHC plate used to validate the developed model.

To verify the accuracy and reliability of the proposed method, a verification example of the cantilever SMAHC plate, as shown in Figure 3, is investigated based on the developed finite element code in Matlab. The SMAHC plate consists of four graphite/epoxy plies and one surface-mounted SMAHC layer. The material properties of graphite/epoxy lamina are as follows: *E*_11_ = 138 GPa, *E*_22_ = *E*_33_ = 8.28 GPa, *ν*_12_ = *ν*_13_ = 0.33, *ν*_23_ = 0.37, *G*_12_ = *G*_13_ = 6.9 GPa, *G*_23_ = 8.28 GPa, *ρ* = 1600 kg/m^3^, *α*_1_ = 0.18 × $10^{-6}$ (K) and *α*_2_ = *α*_3_ = 27.0 × $10^{-6}$ (K). The material properties of the SMA layer are listed in Table 1. The geometric dimensions and the loading of the numerical example are shown in Figure 2. The verification computations were carried out for the following three different stacking sequences: case I, [0/0/0/0/0]_Gr/E_, [SMAHC/0/0/0/0]_hybrid_; case II, [45/−45/45/−45/45]_Gr/E_, [SMAHC/−45/45/−45/45]_hybrid_; and case III, [90/0/90/0/90]_Gr/E_, [SMAHC/0/90/0/90]_hybrid_. The SMAHC contains 20% volume fraction of SMA wire, and they are considered to be evenly distributed along the layer width.

Table 2 presents the static deflections and first three natural frequencies of traditional graphite/epoxy laminates and the SMAHC plate, and also the comparison with the results obtained in Ref. [35] based on a three-dimensional shell finite element method. The computations were performed at a constant temperature of 5 °C. It should be noted that the FEM results are obtained with a uniform mesh of 4 × 4 eight-node quadratic element (4Q8) based on FDST. Then, an equal quadratic Lagrange interpolation functions of rectangular elements interpolation is employed for all generalized displacements, so that the number of degrees of freedom per element is 40. For the selected mesh, the integration rule is 3 × 3 Gauss rule.

It is evident that both the static deflection and natural frequency results obtained from the presented method are in good agreement with the previously published results [35]. Comparing the results between the SMAHC plate and graphite/epoxy plate, the SMAHC plate possesses a higher deflection and a lower natural frequency due to the lower stiffness of SMAHC lamina. As expected, the first layup, [0/0/0/0/0]_Gr/E_, [SMAHC/0/0/0/0]_hybrid_, has the greatest stiffness. In contrast, the third layup, [45/−45/45/−45/45]_Gr/E_, [(SMA/E)/−45/45/−45/45]_hybrid_, shows the smallest natural frequency and the largest static deflection.

**Figure 4:**
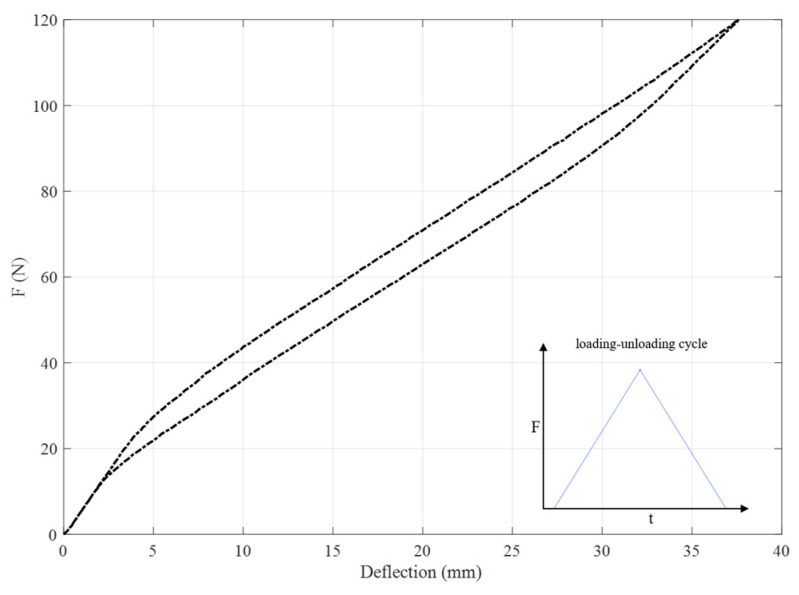
Tip force vs. vertical deflection of the hybrid plate ([(SMAHC)/0/0/0/0]) during a full loading–unloading cycle.

Moreover, the mechanical response of the hybrid plate ([(SMAHC)/0/0/0/0]) obtained by the presented method is shown in Figure 4 in terms of the free end force vs. the vertical deflection under a complete loading-unloading cycle. Figure 4 shows that the obtained force-deflection curve shows a typical hysteresis behavior which can be applied to dampen vibrations.

### 3.2. Validation of Developed Model (Parametric Investigations)

In this section, a detailed parametric study is carried to investigate the influence of various parameters on the thermal vibrations of the SMAHC plate. In this case study, a six-layered hybrid plate of 100 × 100 × 6 mm is simulated. The hybrid plate consists of four glass fiber/epoxy laminas and two surface-mounted NiTi wire-reinforced glass fiber/epoxy hybrid layers. The hybrid plate layup and coordinates are illustrated in Figure 1. The SMA wires are assumed to be embedded along the *y* direction and uniformly distributed along the layer width. The material properties of glass fiber/epoxy lamina are as follows: *E*_11_ = 40 GPa, *E*_22_ = *E*_33_ = 10 GPa, *ν*_12_ = *ν*_13_ = 0.25, *G*_12_ = *G*_13_ = 5 GPa, *G*_23_ = 2 GPa, *ρ* = 1850 kg/m^3^, α_1_ = 6.3 × $10^{-6}$(K) and α_2_ = α_3_ = 20 × $10^{-6}$(K). We remind the reader that the material properties of the SMA layer are summarized in Table 1.

**Figure 5:**
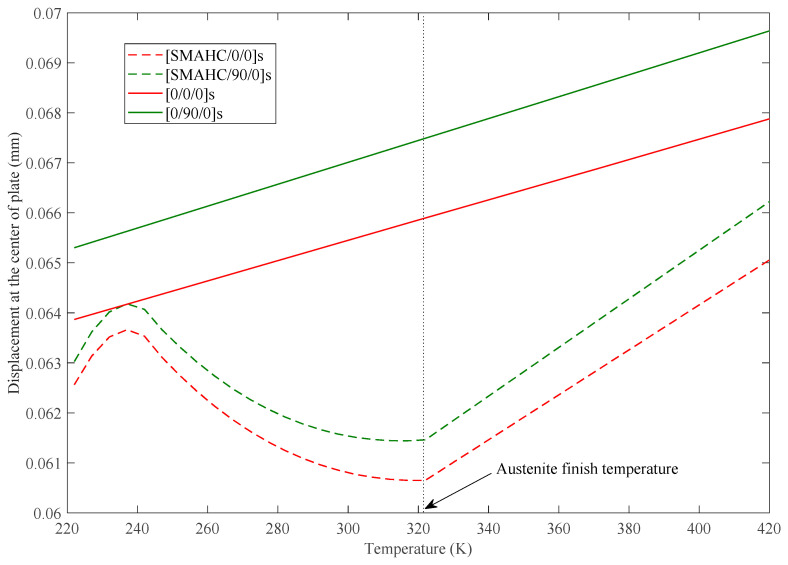
Static deflection at the center of the plate vs. temperature, with *Vs* = 30%, *ε* = 5%, CCCC boundary condition.

**Figure 6:**
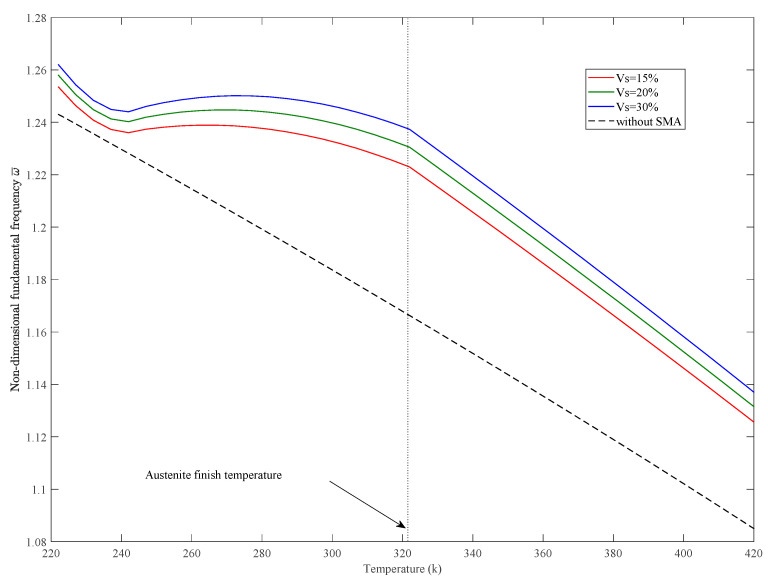
Variation of nondimensional fundamental frequency with temperature for different SMA volume fractions *Vs*, with [SMAHC/0/0/0]s, *ε* = 5%, CCCC boundary condition.

**Figure 7:**
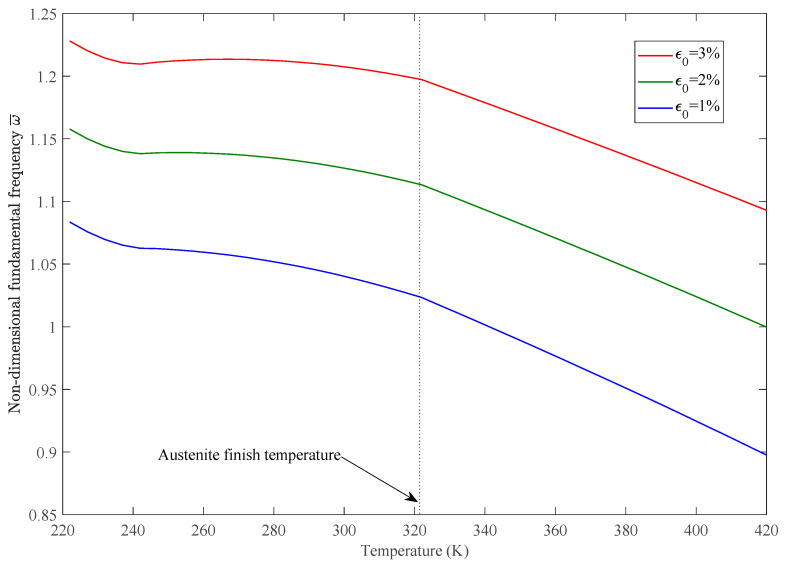
Variation of nondimensional fundamental frequency with temperature for different SMA pre-strains, with [SMAHC/0/0/0]s, *Vs* = 15%, CCCC boundary condition.

**Figure 8:**
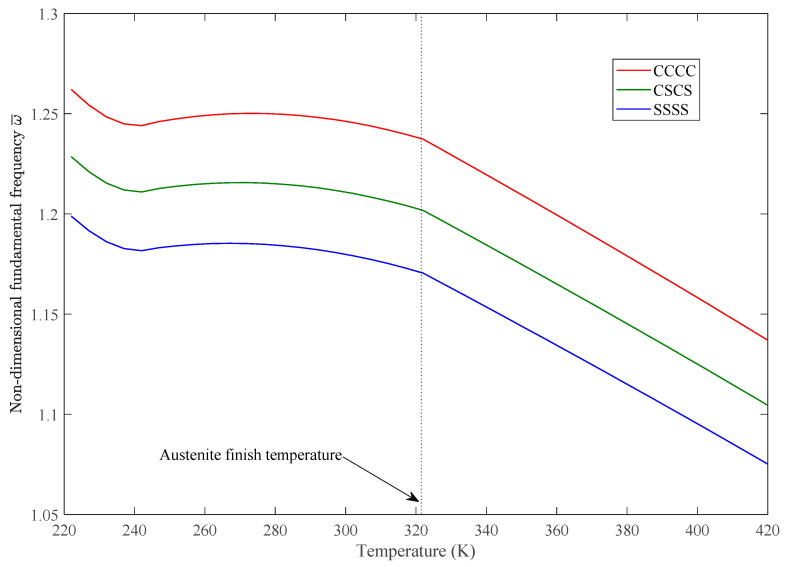
Variation of nondimensional fundamental frequency with temperature for different boundary conditions, with [SMAHC/0/0/0]s, *Vs* = 15%, *ε* = 5%.

**Figure 9:**
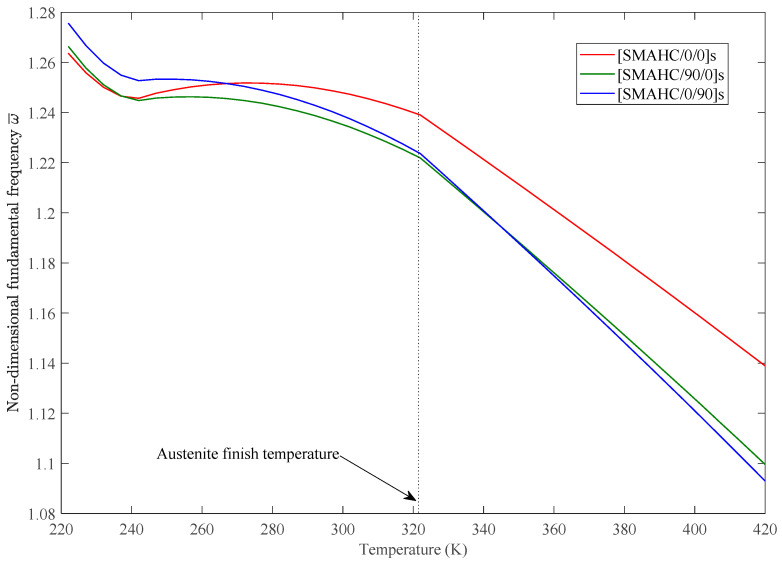
Variation of nondimensional fundamental frequency with temperature for different stacking sequences, with *Vs* = 30%, *ε* = 5%, CCCC boundary condition.

The variations of static deflection and nondimensional fundamental frequencies $\bar{w}=w*\left(ab/h\right)*{\left(\rho /E_{1}\right)}_{Gl/E}^{1/2}\;$in thermal environments of the SMAHC plate are presented in Figure 5, Figure 6, Figure 7, Figure 8 and Figure 9. It is evident from the presented results that the variations of both the static deflection and natural frequency of the SMAHC plate over a temperature show a non-uniform behavior. Present results show that the stiffness of the SMAHC plate initially decreases, followed by a gradual increase due to the recovery stress of the SMA wires, and finally decreases for all the cases. On the contrary, for the conventional plates without SMA wires, the variation of structural stiffness with temperature shows a uniform decrease. It should be noted that SMAHC plate vibrational behaviors are highly dependent on both the thermal stress induced by thermal expansion and the phase transformations of SMA wires. It can be seen from all plots that when the SMAHC plate has a temperature lower than austenite finish temperature A_f_, the variations of the vibrational frequencies with temperature are more complicated because the structural stiffness is affected by the thermally induced stress and a phase transformation in SMA. However, at the temperature of A_f_ or above, the entire SMAHC plate becomes austenite, and correspondingly, the thermal stress induced by thermal expansion becomes the only parameter that affects the variation of the natural frequency with temperature.

More specifically, for static response analysis, a uniform load of 1.25 kN is applied on the upper surface of the plate. The effect of temperature and stacking sequence on the plate static deflection is presented in Figure 5. The results show the considerable effect of temperature on the variation of static deflection for both plates (i.e., with and without SMA wires). As evident in Figure 5, the static deflection of the SMAHC plate shows the multistage variation caused by a phase transformation of SMA wires, while the conventional composite plate exhibits only a monotonically increasing behavior with temperature.

Figure 6 presents the influence of SMA wire volume fraction Vs on the fundamental natural frequency under thermal environment together with the results of the conventional glass fiber/epoxy plate without SMA wires. Briefly, by increasing the SMA wire volume fraction, the fundamental frequency is accordingly increased. In addition, the natural frequencies of the SMAHC plate are higher than those obtained for conventional plates.

The effect of SMA wire pre-strain value on thermal vibration is shown in Figure 7. Here, an increased value of pre-strain of SMA wire shifts the natural frequency to higher values. This increase is caused by the higher SMA pre-strain that leads to higher stiffness of SMAHC lamina.

Figure 8 shows the effect of boundary conditions on the natural frequency of the SMAHC plate. It has been determined that the boundary condition has a significant effect on the vibrational behavior of the SMAHC plate. Three edge support conditions are studied in this work—CCCC, CSCS and SSSS—in which C and S represent clamped and simply supported boundary conditions, respectively. As expected, the SMAHC plate with CCCC boundary conditions has a higher natural frequency, and the lowest natural frequencies belong to the plate with *SSSS* boundary conditions.

Results summarized in Figure 9 present the effect of stacking sequence on the natural frequency of the SMAHC plate. It is observed that SMAHC plates with different stacking sequences have remarkably different variations according to different temperature zones. When the temperature is lower than austenite start temperature *A*s, the [SMAHC/0/0]s layup has the lowest natural frequency. However, the slope of curvature of [SMAHC/0/0]s layup gradually decreases during the austenite phase transformation, which leads to the highest natural frequency (i.e., when temperature is above austenite finish temperature A_f_). In contrast, the [SMAHC/0/90]s layup varies in the opposite way.

## 4. Conclusions

In this study, a coupled thermoelastic FEM for static and free vibration analysis of hybrid composite plates with embedded SMA wires was developed. The model was derived based on the FSDT and Brinson’s constitutive model for SMA, which accounts for both the transformation phases of SMA and thermal environment. The results obtained from the proposed model were validated by comparison with predictions from previously published data. Moreover, based on the proposed method, a parametric analysis was investigated, and the following conclusions can be drawn:

1. The results of thermal static deflection and free vibration indicate that by embedding SMA wire into conventional composite laminates, the load-carrying capacity can be significantly increased under a thermal environment. The results can be implemented for the active property tuning (APT) application, where the stiffness of the host structure can be modified by the change in the Young’s modulus of SMA wires with heating.
2. Thermal frequencies of SMAHC plates exhibit a non-uniform behavior, whereas thermal frequencies of composite plates without SMA decrease with increasing temperature.
3. The SMAHC plate has higher structural stiffness, which leads to higher thermal frequencies and lower static deflections compared to the results from conventional composite plates.
4. By increasing the SMA volume fraction and pre-strain, the stiffness of the SMAHC plate is increased accordingly. These results can be employed for the active strain energy tuning (ASET) application, where the generation of high recovery stresses within the host structure can be implemented by preparing initial strains before embedding the SMA wires into a composite medium.
5. SMAHC plates with different stacking sequences exhibit significant thermal vibration behaviors at different temperature zones. For design purposes, to ensure better structural performances in thermal environment, it is recommended to use an SMAHC plate with the proper stacking sequence according to its service temperatures.

In summary, the proposed finite element model is capable of predicting thermal-mechanical coupling behavior of SMAHC plates. However, in order to achieve a more accurate and effective simulation, recommendations for a possible future study are listed as follows:

1. Geometrical nonlinearities should be considered for simulation of large strain recoverability of SMA elements.
2. A more realistic description of the kinematics of composite laminates, such as higher-order shear deformation theory or layer-wise theory, is recommended in order to accurately determine the stress state at the layer level.

## Figures and Tables

**Table 1:** Considered properties of SMA.

Property	Variable	Value
The Young’s modulus (austenite)	*E*_A_	67 GPa
The Young’s modulus (martensite)	*E*_M_	26.3 GPa
Austenite start temperature	*A_s_*	34.5 °C
Austenite finish temperature	*A_f_*	49 °C
Martensite start temperature	*M_s_*	18.4 °C
Martensite finish temperature	*M_f_*	9 °C
Critical transformation stress	$\sigma_{s}^{cr}$, $\sigma_{f}^{cr}$	100 MPa, 170 MPa
Slope of martensite limit curve	*C*_M_	8.0 MPa/°C
Slope of austenite limit curve	*C*_A_	13.8 MPa/°C
Maximum transformation strain	$\varepsilon_{L}$	0.067
Density	$\rho_{s}$	6648.1 kg/m^3^
Thermal expansion	$\alpha_{A}$, $\alpha_{M}$	11 × $10^{-6}$ (°C), 6.6 × $10^{-6}$ (°C)

**Table 2:** Comparison of the deflections and natural frequencies of Gr/E and SMAHC plates.

Configuration	Obtained Property	Gr/E (Ref. [35]/Present Study)	SMAHC Plate (Ref. [35]/Present Study)
I. layup	Deflection (mm) Natural frequency (Hz)	37.6/37.4	65.0/64.7
		52.2/52.3	39.4/39.9
		86.4/87.1	63.2/64.5
		231.7/232.8	167.2/169.4
II. layup	Deflection (mm) Natural frequency (Hz)	163.7/163.3	288.8/288.5
		25.7/25.7	19.3/19.4
		123/123.6	97.5/98.2
		157.3/158.9	116/117.3
III. layup	Deflection (mm) Natural frequency (Hz)	146.2/146	160.9/160.4
		26.4/26.9	25.1/25.4
		69.8/70.7	54.1/55.2
		165.5/166.2	157.5/159.3

## Data Availability

Not applicable.

## Appendix A

Detailed kinetic equations of phase transformation are expressed by using the empirical cosine model of Brinson (see Ref. [39]), as follows:

(a.) Phase transformation to martensite: If $T>M_{s}\;$ and $\sigma_{s}^{cr}+C_{M}\left(T-M_{s}\right)<\sigma <\sigma_{f}^{cr}+C_{M}\left(T-M_{f}\right)$ then:
  (A1) $$\xi_{s}=\frac{1+\xi_{s0}}{2}+\frac{1-\xi_{s0}}{2}\cos\left\{\frac{\pi }{\sigma_{s}^{cr}-\sigma_{f}^{cr}}\left(\sigma -\sigma_{f}^{cr}-C_{M}\left(T-M_{s}\right)\right)\right\}$$
  and
  (A2) $$\xi_{T}=\xi_{T0}-\frac{\xi_{T0}}{1-\xi_{s0}}\left(\xi_{s}-\xi_{s0}\right)$$
  If $T<M_{s}\;$and $\sigma_{s}^{cr}<\sigma <\sigma_{f}^{cr}$ then:
  (A3) $$\xi_{s}=\frac{1+\xi_{s0}}{2}+\frac{1-\xi_{s0}}{2}\cos\left\{\frac{\pi }{\sigma_{s}^{cr}-\sigma_{f}^{cr}}\left(\sigma -\sigma_{f}^{cr}\right)\right\}$$
  and
  (A4) $$\xi_{T}=\xi_{T0}-\frac{\xi_{T0}}{1-\xi_{s0}}\left(\xi_{s}-\xi_{s0}\right)+\Delta_{T\xi },$$
  where $\Delta_{T\xi }=\{\begin{array}{cc} \frac{1-\xi_{T0}}{2}\left\{\cos\left(C_{M}\left(T-M_{f}\right)\right)+1\right\}, & \;if\;M_{f}<T<M_{s}\;and\;T<T_{0}\\ 0, & else \end{array}$
(b.) Phase transformation to austenite: If $T>A_{s}\;$ and $C_{A}\left(T-A_{f}\right)<\sigma <C_{A}\left(T-A_{s}\right)$ then:
  (A5) $$\xi =\frac{\xi_{0}}{2}\left\{\cos\left(\frac{\pi }{A_{f}-A_{s}}\left(T-A_{s}-\frac{\sigma }{C_{A}}\right)\right)+1\right\},$$
  (A6) $$\xi_{s}=\xi_{s0}-\frac{\xi_{s0}}{\xi_{0}}\left(\xi_{0}-\xi \right),$$
  (A7) $$\xi_{T}=\xi_{T0}-\frac{\xi_{T0}}{\xi_{0}}\left(\xi_{0}-\xi \right).$$

## Appendix B

The thermo-mechanical properties of an SMA wire-reinforced hybrid composite lamina can be determined by using a general micro/macro mixture method. The elastic properties of an SMAHC lamina are given as:

(A8) $$E_{11}=E_{s}V_{s}+E_{m}\left(1-V_{s}\right),$$

(A9) $$E_{22}=E_{33}=\frac{E_{m}}{1-\sqrt{V_{s}}\left(1-E_{m}/E_{s}\right)},$$

(A10) $$G_{12}=G_{13}=\frac{G_{m}}{1-\sqrt{V_{s}}\left(1-G_{m}/G_{s}\right)}.$$

(A11) $$G_{23}=\frac{G_{m}}{1-V_{s}\left(1-G_{m}/G_{s}\right)}.$$

The density is given by:

(A12) $$\rho =\rho_{s}V_{s}+\rho_{m}\left(1-V_{s}\right).$$

The Poisson’s ratio is given by:

(A13) $$\nu_{12}=\nu_{13}=\nu_{s}V_{s}+\nu_{m}\left(1-V_{s}\right),$$

(A14) $$\nu_{23}=\nu_{s}V_{s}+\left(1-V_{s}\right)\left(2\nu_{m}-\frac{\nu_{12}E_{22}}{E_{11}}\right).$$

Finally, the coefficient of thermal expansion of SMAHC lamina can be defined as:

(A15) $$\alpha_{1}=\frac{\alpha_{s}E_{s}V_{s}+\alpha_{m}E_{m}\left(1-V_{s}\right)}{E_{11}},$$

(A16) $$\alpha_{2}=\frac{E_{m}}{E_{22}}\left[\alpha_{m}\left(1-\sqrt{V_{s}}\right)+\frac{\alpha_{m}\sqrt{V_{s}}-V_{s}\left(\alpha_{m}-\alpha_{s}\right)}{1-\sqrt{V_{s}}\left(1-E_{m}/E_{s}\right)}\right].$$

where the subscript “*s*” and “*m*” indicate the SMA material and composite matrix material, respectively.

## Appendix C

Expressions of submatrices listed in Equation (19) are given as follows:

(A17) $$\left[\begin{array}{ccc} M_{0} & M_{1} & M_{2} \end{array}\right]=\int_{\Omega_{e}}^{}\left[\begin{array}{ccc} I_{0} & I_{1} & I_{2} \end{array}\right]\psi_{i}^{e}\psi_{j}^{e}dxdy,$$

(A18) $$\left[\begin{array}{cc} \begin{array}{cc} F^{1} & F^{2} \end{array} & \begin{array}{cc} F^{4} & F^{5} \end{array} \end{array}\right]=\oint_{\Gamma_{e}}^{}\left[\begin{array}{cc} \begin{array}{cc} P_{x} & P_{y} \end{array} & \begin{array}{cc} T_{x} & T_{y} \end{array} \end{array}\right]\psi_{i}^{e}dxdy,$$

(A19) $$F^{3}=\int_{\Omega_{e}}^{}q_{z}\psi_{i}^{e}dxdy\;dxdy,$$

(A20) $$F^{T1}=\oint_{\Gamma_{e}}^{}\left(\psi_{i,x}^{e}N_{xx}^{T}+\psi_{i,y}^{e}N_{xy}^{T}\right)dxdy\;dxdy,$$

(A21) $$F^{T2}=\oint_{\Gamma_{e}}^{}\left(\psi_{i,x}^{e}N_{xy}^{T}+\psi_{i,y}^{e}N_{yy}^{T}\right)dxdy\;dxdy,$$

(A22) $$F^{T4}=\oint_{\Gamma_{e}}^{}\left(\psi_{i,x}^{e}M_{xx}^{T}+\psi_{i,y}^{e}M_{xy}^{T}\right)dxdy\;dxdy,$$

(A23) $$F^{T5}=\oint_{\Gamma_{e}}^{}\left(\psi_{i,x}^{e}M_{xy}^{T}+\psi_{i,y}^{e}M_{yy}^{T}\right)dxdy\;dxdy,$$

(A24) $$F^{r1}=\oint_{\Gamma_{e}}^{}\left(\psi_{i,x}^{e}N_{xx}^{r}+\psi_{i,y}^{e}N_{xy}^{r}\right)dxdy\;dxdy,$$

(A25) $$F^{r2}=\oint_{\Gamma_{e}}^{}\left(\psi_{i,x}^{e}N_{xy}^{r}+\psi_{i,y}^{e}N_{yy}^{r}\right)dxdy\;dxdy,$$

(A26) $$F^{r4}=\oint_{\Gamma_{e}}^{}\left(\psi_{i,x}^{e}M_{xx}^{r}+\psi_{i,y}^{e}M_{xy}^{r}\right)dxdy\;dxdy,$$

(A27) $$F^{r5}=\oint_{\Gamma_{e}}^{}\left(\psi_{i,x}^{e}M_{xy}^{r}+\psi_{i,y}^{e}M_{yy}^{r}\right)dxdy\;dxdy.$$

Here:

$$\begin{array}{c} P_{x}=N_{xx}n_{x}+N_{xy}n_{y}\\ P_{y}=N_{xy}n_{x}+N_{yy}n_{y}\\ T_{x}=M_{xx}n_{x}+M_{xy}n_{y}\\ T_{y}=M_{xy}n_{x}+M_{yy}n_{y} \end{array}$$

where $\left(n_{x},\;n_{y}\right)$ are the direction cosines of the unit normal on the boundary Γ.
